# Cell Expansion-Dependent Inflammatory and Metabolic Profile of Human Bone Marrow Mesenchymal Stem Cells

**DOI:** 10.3389/fphys.2016.00548

**Published:** 2016-11-16

**Authors:** Patricia Prieto, María Fernández-Velasco, María E. Fernández-Santos, Pedro L. Sánchez, Verónica Terrón, Paloma Martín-Sanz, Francisco Fernández-Avilés, Lisardo Boscá

**Affiliations:** ^1^Instituto de Investigaciones Biomédicas Alberto Sols (CSIC-UAM)Madrid, Spain; ^2^Instituto de Investigación Hospital Universitario La PazMadrid, Spain; ^3^Servicio de Cardiología, Hospital General Universitario Gregorio Marañón, Instituto de Investigación Sanitaria Gregorio MarañónMadrid, Spain; ^4^Servicio de Cardiología, Hospital Clínico de SalamancaSalamanca, Spain; ^5^Facultad de Medicina, Universidad Complutense de Madrid, Ciudad UniversitariaMadrid, Spain

**Keywords:** inflammation, cytokines, human, stem cell, bone marrow

## Abstract

Stem cell therapy has emerged as a promising new area in regenerative medicine allowing the recovery of viable tissues. Among the many sources of adult stem cells, bone marrow-derived are easy to expand in culture *via* plastic adherence and their multipotentiality for differentiation make them ideal for clinical applications. Interestingly, several studies have indicated that MSCs expansion *in vitro* may be limited mainly due to “cell aging” related to the number of cell divisions in culture. We have determined that MSCs exhibit a progressive decline across successive passages in the expression of stem cell markers, in plasticity and in the inflammatory response, presenting low immunogenicity. We have exposed human MSCs after several passages to TLRs ligands and analyzed their inflammatory response. These cells responded to pro-inflammatory stimuli (i.e., NOS-2 expression) and to anti-inflammatory cytokines (i.e., HO1 and Arg1) until two expansions, rapidly declining upon subculture. Moreover, in the first passages, MSCs were capable to release IL1β, IL6, and IL8, as well as to produce active MMPs allowing them to migrate. Interestingly enough, after two passages, anaerobic glycolysis was enhanced releasing high levels of lactate to the extracellular medium. All these results may have important implications for the safety and efficacy of MSCs-based cell therapies.

## Introduction

Mesenchymal stem cells (MSCs) are multipotent adult stem cells capable to differentiate into mesenchymal-type cells (adipocytes, osteoblasts, and chondrocytes) but also myocytes, neurons, endothelial cells, astrocytes and epithelial cells (Woodbury et al., 2002). These cells can be obtained from bone marrow, umbilical cord blood, peripheral blood and adipose tissue (DelaRosa and Lombardo, 2010). Nowadays, MSCs are an interesting tool for cell therapy and tissue repair because they can be activated and recruited to sites of tissue damage where they regenerate new tissues. Clinical applications require their expansion and differentiation *in vitro* before using in patients to obtain an adequate number of cells. Despite the efforts that have been made, there are several problems associated to MSCs culture *in vitro* before using them in regeneration therapies (Ikebe and Suzuki, 2014). Initially, bone-marrow MSCs emerged as useful candidates due to their potent self-renewal capacity. However, further studies of MSCs populations indicated that *in vitro* expansion may be limited by problems associated to their prolonged culture (Wei et al., 2013). Since self-renewal is the process by which stem cells proliferate and generate more stem cells, during the passages these cells accumulate mutations in their genome with every round of DNA replication. This fact remains controversial because MSCs are capable to form colonies without maintenance of their initial multipotentiality. Therefore, the determination of the number of replications that can be done *in vitro* of MSCs before losing their “stemness” properties is essential to achieve any success in future clinical applications.

MSCs are being used in several clinical trials, mainly because of their “low immunogenic” properties (Wei et al., 2013). Accordingly, *ex vivo* expanded MSCs have been reported to inhibit activation, proliferation and function of immune cells through cell contact mechanisms and by soluble factors secreted by MSCs in response immune cells cross-talk (Di Nicola et al., 2002; Zhang et al., 2004; Corcione et al., 2006). Interestingly, bone marrow-derived MSCs express several TLRs, highlighting the presence of functional TLR2, TLR3, and TLR4 which can be regulated by several cellular contexts, such as hypoxic conditions (Mo et al., 2008) or viral transduction (Chen et al., 2009). Moreover, engagement of these receptors with specific ligands resulted in the activation of signaling pathways (i.e., MAPKs and PI3K) and transcription of NF-κB-dependent genes, such as COX-2, IL6, and IL8 (Lombardo et al., 2009). Nevertheless, the response of MSCs to inflammatory stimuli remained to be determined. Thus, is has become essential the understanding of the immune behavior of these cells *in vitro*.

Another interesting but less understood aspect of MSCs is their ability to migrate from bone marrow or peripheral blood into damaged tissues. A key requirement for cells to reach distant target sites is the ability to traverse the extracellular matrix which is present between cells of all tissue types (Kalluri, 2003). Migrating cells need to express proteolytic enzymes known as matrix metalloproteinases (MMPs), which are involved in many physiological or pathological conditions, being MMP2, and MMP9 the most important in MSC context (Visse and Nagase, 2003; Koippallil Gopalakrishnan Nair et al., 2015). The biosynthesis and activity of MMPs are associated with the invasive capacity of various cell types (Egeblad and Werb, 2002). These proteins are secreted in cells as inactive zymogens which are rapidly complexed by their specific endogenous inhibitors, the tissue inhibitors of metalloproteinases (TIMPs). It has been described that both TIMP1 and TIMP2 can be secreted by MSCs promoting an anti-metastatic phenotype (Itoh et al., 2001; Clarke et al., 2015). Thus, as the balance of MMP/TIMP could modulate the migratory capacity of MSC, is it important to determine if there is some kind of modulation along the passages. If MSCs are unable to reach the target tissue, then these cells lose their potential application in regenerative medicine.

Another key factor is the stability of the metabolic profile of MSCs after successive replications. Recent studies have revealed a shift in the balance between glycolysis, mitochondrial oxidative phosphorylation and oxidative stress during the maturation of adult stem cells, and during the reprogramming of somatic cells to pluripotency (Pattappa et al., 2011). Thus, it is necessary to define and ensure the “stemness” properties of these human cells upon expansion. These insights shed light to the controversial use of MSCs to enhance regeneration and the treatment of degenerative diseases.

## Results

**Figure 1:**
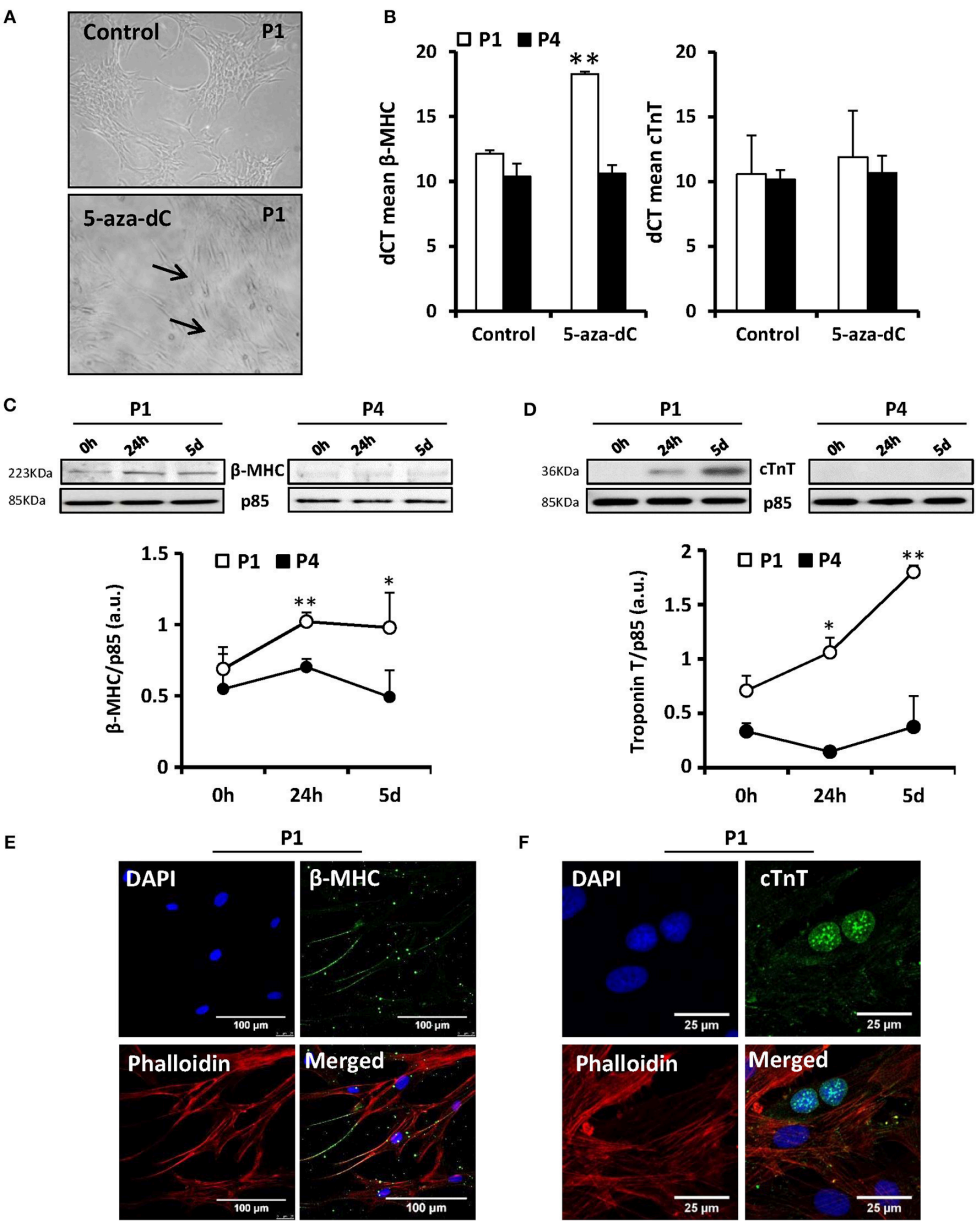
**MSCs exposure to 5′-aza-2′-deoxycytidine (5-aza-dC) induces *in vitro* differentiation to a “myogenic phenotype” in P1 (first passage) but this effect is lost at P4**. The morphology of MSCs changed after 5-aza-dC exposure from fibroblast-like to form a stick-like morphology and several primitive myofilaments were detected in P1 **(A)**. The mRNA levels (qPCR) of the specific marker cardiac myosin (β-MHC) were significantly augmented after 5-aza-dC treatment in P1 without changes in cardiac troponin (cTnT) levels. No changes were detected in P4 **(B)**. The expression of β-MHC and cTnT was enhanced at the protein level (Western blot; **C,D**) and by immunofluorescence analysis, using DAPI (nuclear) and phalloidin (actin) staining **(E,F)**, after 5-aza-dC in P1 but was missing in P4. ^*^p < 0.05; ^**^p < 0.01 vs. the untreated cells (0 h).

**Figure 2:**
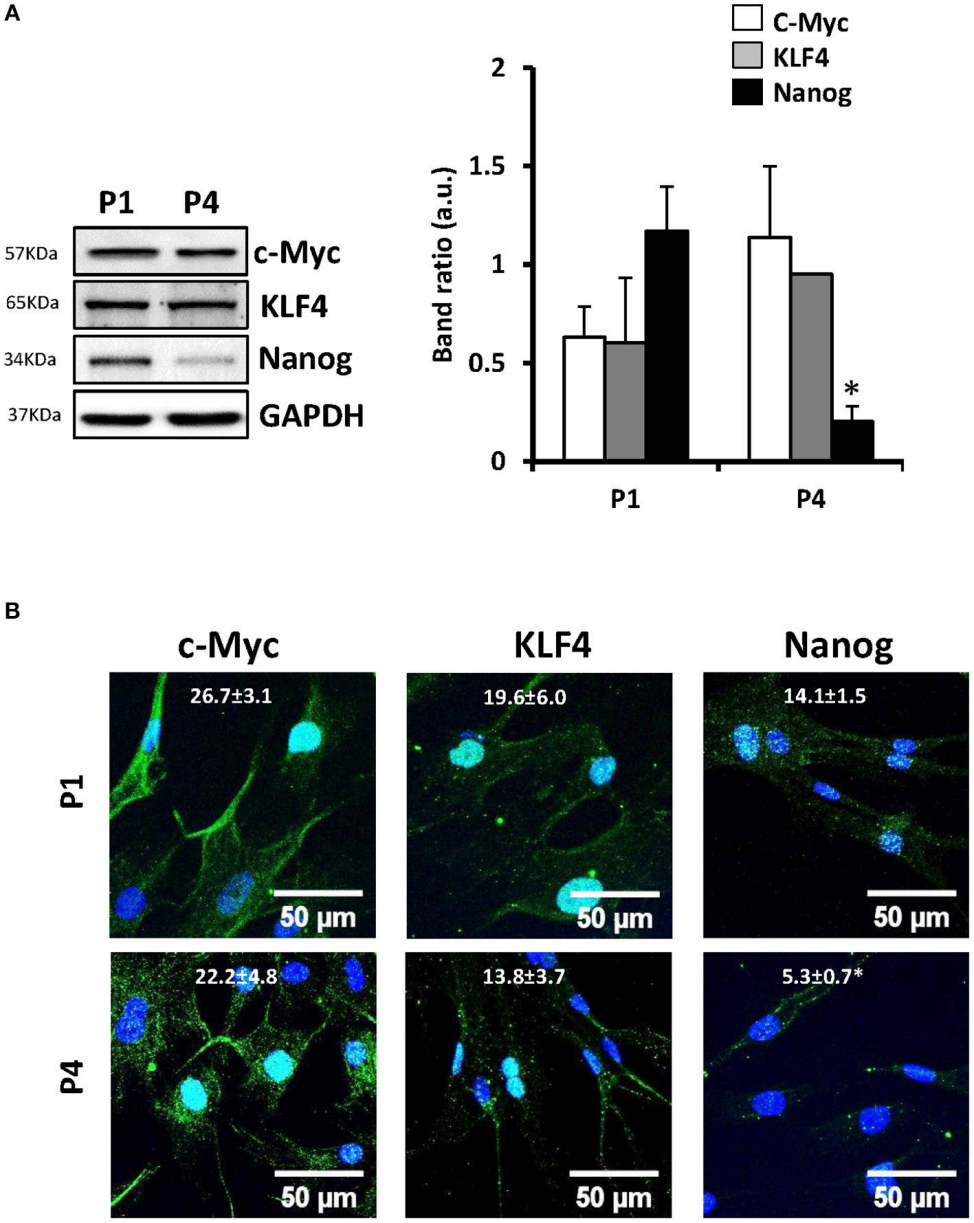
**The expression of some “stem cell markers” decays in MSCs after prolonged number of replications *in vitro***. MSCs were maintained in culture and the protein levels of the “stem cell markers” c-Myc, KLF4 and Nanog was analyzed by Western blot **(A)** and by immunofluorescence (nuclei stained with DAPI, **B**). Values are the mean ± SD of the band ratios **(A)** or the green fluorescence **(B)**. ^*^p < 0.05; vs. P1 cells.

The main interest to study MSCs is their promising role in tissue regeneration because of their multipotentiality. However, one of the main problems associated to MSCs is that they suffer genetic alterations after several divisions *in vitro* augmenting their genomic instability (Ross et al., 2011). Therefore, prior to widespread use of these cells in regenerative medicine, it is important to gain understanding of the specific changes that occur after expansion in successive passages. It has been described that murine or porcine bone marrow-derived MSCs are induced to differentiate into “myogenic cells” *in vitro* with 5-aza-2′-deoxycytidine (5-Aza-dC) treatment (Moscoso et al., 2005). Moreover, MSCs from adult human exposed to this compound are capable to express cardiac-specific genes (Xu et al., 2004). To test this, cells from 11 different donors in culture for 1–4 passages (P1–P4) were exposed to 10 μM of 5-Aza-dC for 24 h or 5 days. After 5 days of 5-Aza-dC treatment, the surviving cells began to proliferate and differentiate. Those of the first passage (P1) exhibited a morphological transition from a fibroblast-like shape to a stick-like appearance exhibiting several primitive myofilaments (Figure 1A); however, this effect disappeared in subsequent passages. We also determined the expression of cardiac markers, such as cardiac myosin (β-MHC) or troponin T (cTnT) by PCR (Figure 1B), Western blot (Figures 1C,D), and immunofluorescence (Figures 1E,F). 5-Aza-dC treatment induced higher levels of both cardiac proteins (at mRNA level also for β-MHC, Figure 1B) at P1 but this effect was missing at P4. Collectively, these results indicated that the capacity of MSCs to underwent cardiomyocyte differentiation in response to 5-Aza-dC treatment rapidly disappear after culture progression. To corroborate the “stemness” phenotype of the MSCs, we analyzed the expression of proteins considered as markers of pluripotency (Wilson et al., 2004; Wang et al., 2013) in all passages. Some of these markers exhibited a rapid drop in protein levels after P1 (Nanog) or maintained a significant expression along passages, such as occurred for KLF4 and c-Myc, probably for this reason cells retained the proliferative capacity (Figures 2A,B). These data suggest that MSCs were capable to maintain their “stemness” properties in culture during the initial fourth passages, being advisable their use within this period for regenerative medicine purposes.

**Figure 3:**
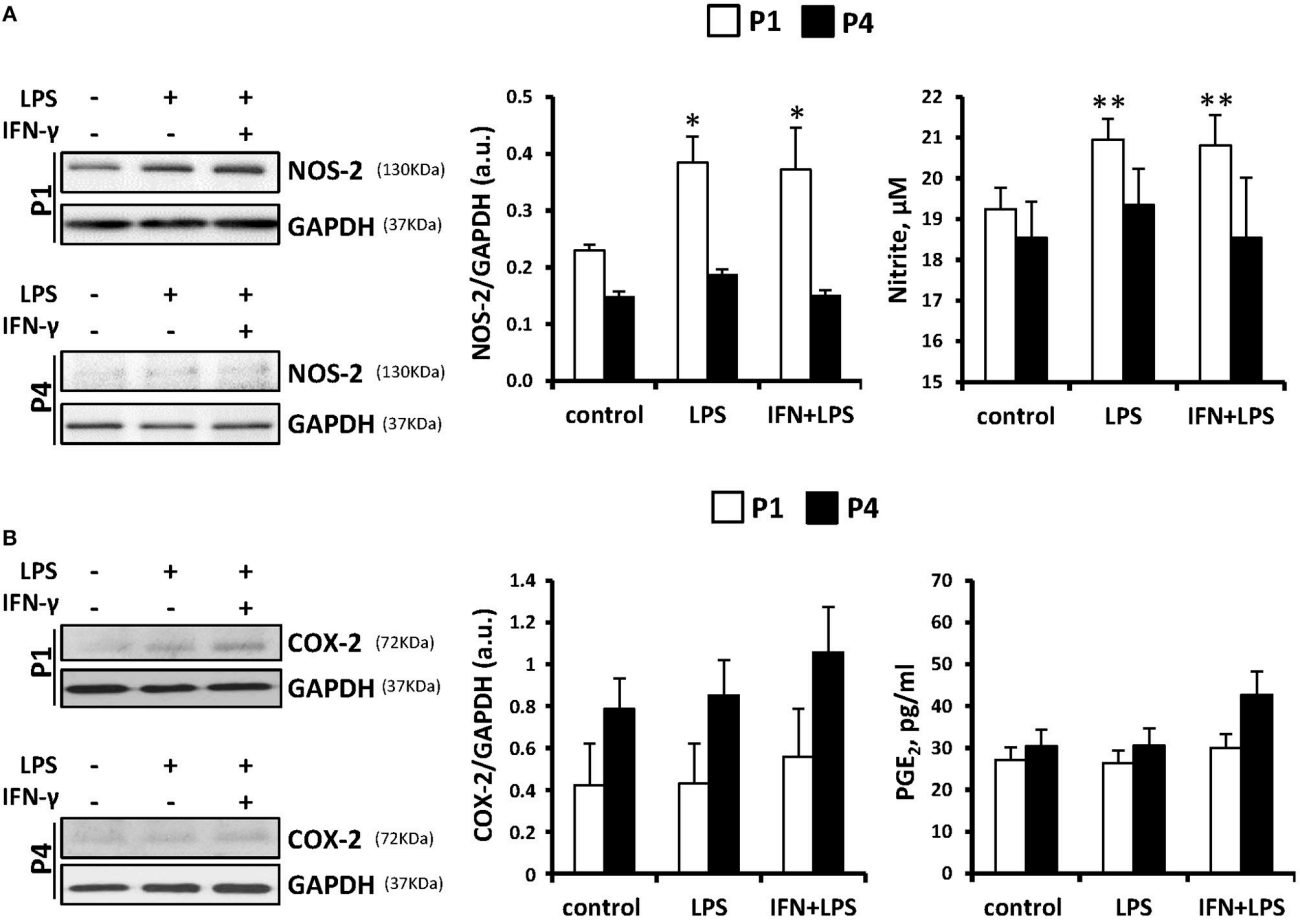
**MSCs treatment with pro-inflammatory stimulus induces the expression of NOS-2 but not COX-2 as well as the accumulation of nitrites in the culture medium**. MSCs were treated with LPS (200 ng/ml) or IFN-γ (20 ng/ml) plus LPS for 24 h as indicated and the protein levels of NOS-2 (**A**, left panel) and COX-2 (**B**, left panel) were analyzed both in P1 and P4. GAPDH was used as normalization protein. The levels of nitrite and PGE_2_ in the culture medium (**A,B**, right panels) were determined at P1 and P4. The histograms show the mean ± SD. ^*^p < 0.05; ^**^p < 0.01 vs. control cells.

**Figure 4:**
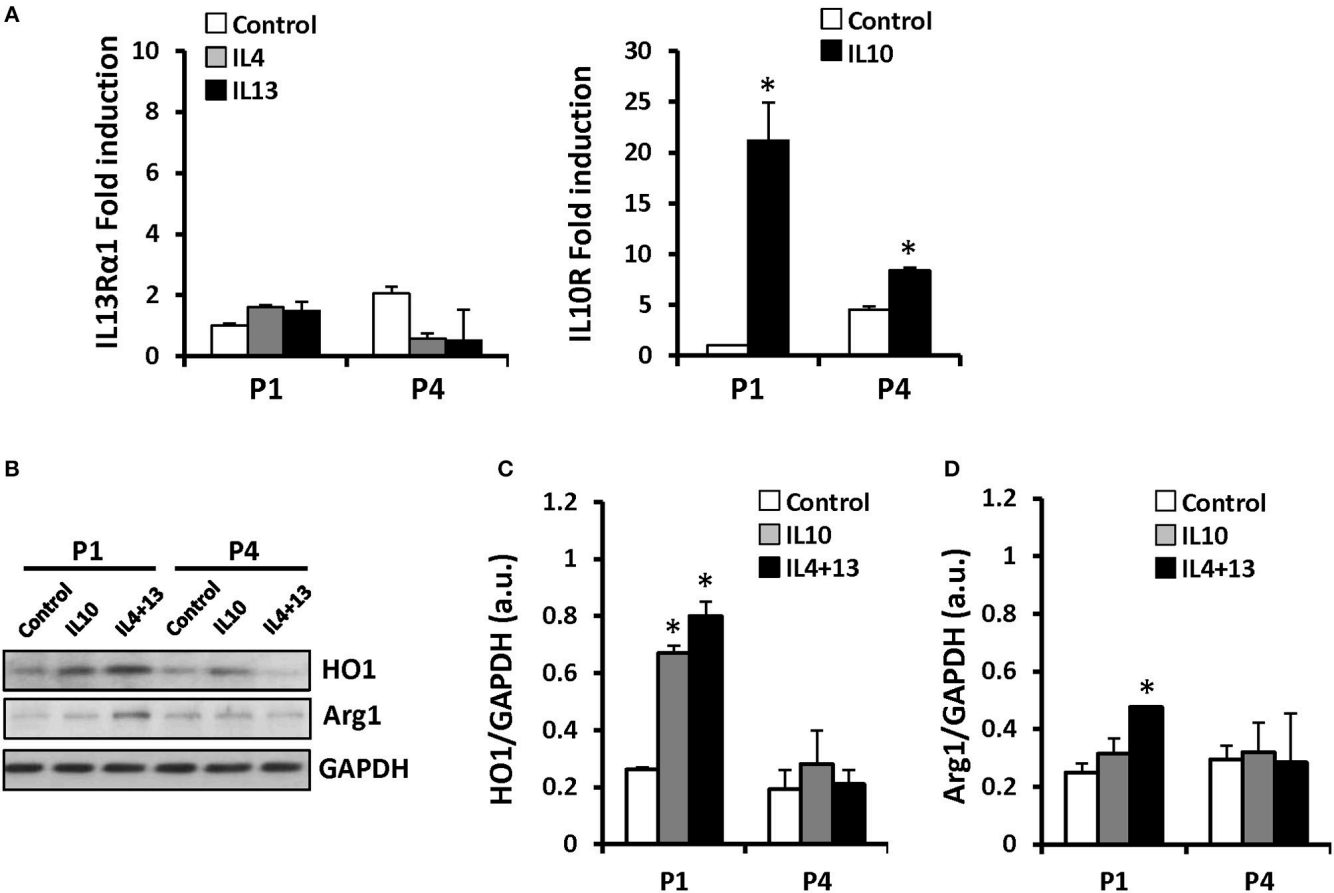
**MSCs treatment with IL10 or IL4+IL13 increases the protein levels of HO1 and Arg1**. MSCs were exposed to IL4, 10, or 13 as indicated and mRNA levels of IL13Rα1 and IL10R were analyzed by qPCR **(A)** after challenge at P1 and P4. HO1 **(B,C)** and Arg1 **(D)** protein levels were induced by IL10 or IL4+IL13 incubation in P1 diminishing in the subsequent. The histograms show the mean ± SD. ^*^p < 0.01 vs. the control condition.

Several groups recognized an immunosuppressive activity for MSC (Ren et al., 2008; Han et al., 2012). However, despite broad research in recent years, whether MSCs are capable to express inflammatory genes in response to pro-inflammatory stimuli remains controversial. We have exposed these cells throughout the successive passages to classical pro- and anti-inflammatory factors and analyzed MSCs responses. Cells treated with LPS (200 ng/ml) or IFN-γ (20 ng/ml) plus LPS showed increased levels of NOS-2 at P1 that rapidly declined in the consecutive expansions. Moreover, nitrite accumulated in the culture medium in agreement to NOS-2 levels (Figure 3A). Interestingly, COX-2 was not significantly increased from P1 and P4 after pro-inflammatory stimulation as well as PGE_2_ levels that remained unchanged (Figure 3B). Furthermore, MSCs express low but constant levels of receptors for IL4 and IL13 (IL13Rα1 subunit as marker; downregulated by IL4 and IL13 at P4) and inducible levels of IL10R that increased after IL10 exposure (Figure 4A), all three cytokines involved in the anti-inflammatory phenotype of immune cells, such as macrophages. Moreover, when MSCs were exposed to IL4 plus IL13 or IL10 (20 ng/ml) and the levels of hemeoxygenase-1 (HO1) and arginase-1 (Arg1) were analyzed, both proteins exhibited a cytokine dependent rise at P1, declining in P4 (Figures 4B–D).

**Figure 5:**
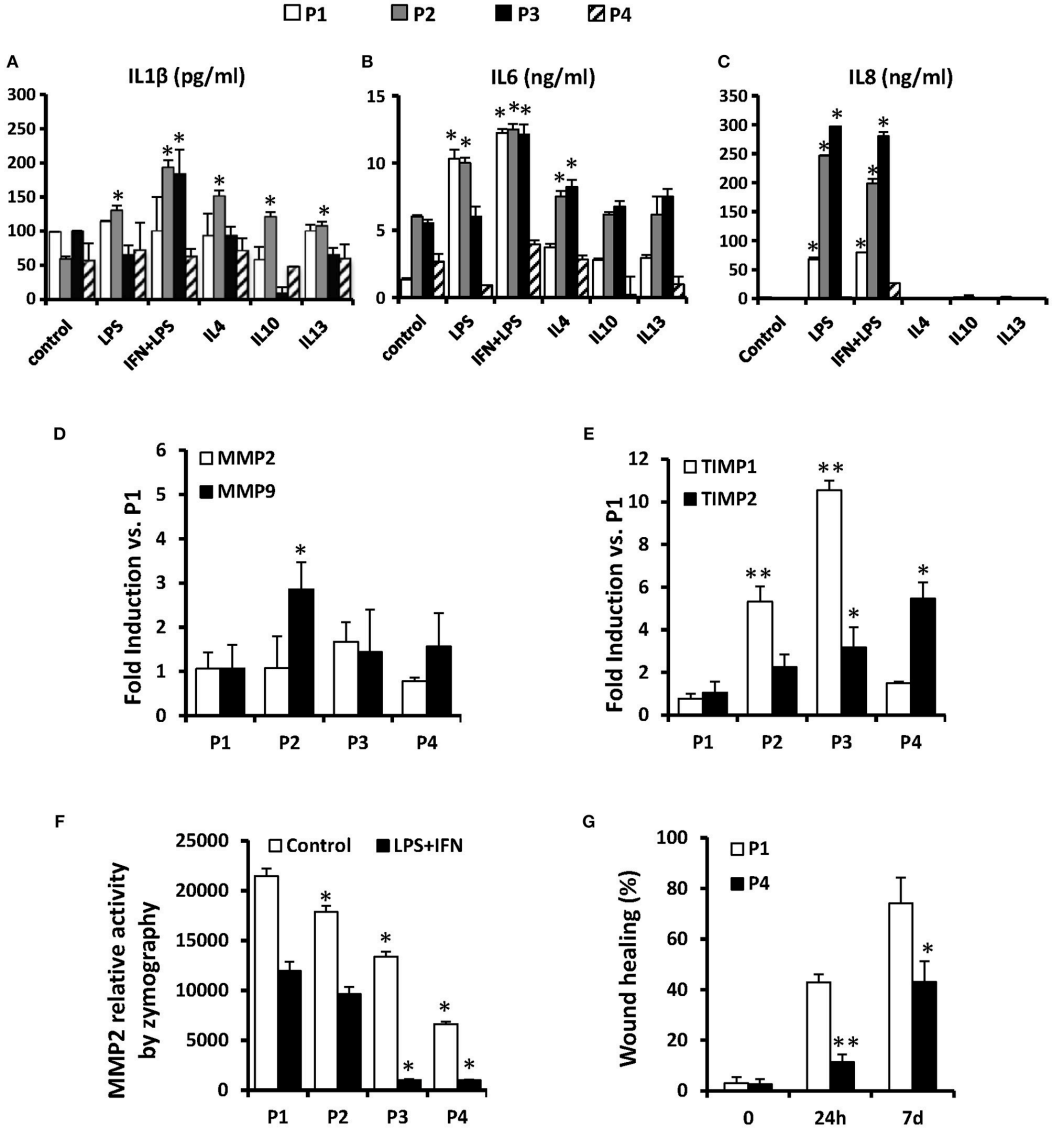
**MSCs release some cytokines *in vitro* such as IL1β, IL6, and IL8 in different inflammatory scenarios and their migration potential is maintained only until P2**. MSCs were treated for 24 h as indicated and the presence of IL1β, IL6, and IL8 were measured in their supernatants **(A–C)**. The mRNA levels of MMP2 and MMP9 **(D)** and of TIMP1 and TIMP2 **(E)** were analyzed after treatments to compare from P1 to P4. The relative activity of MMP2 was analyzed by zymography in gel **(F)** in control and LPS+IFN treated cells from P1 to P4. The migration capacity of cells was measured using a “wound healing” assay **(G)** at 0, 24 h and 7 days in control cells at P1 and P4. ^*^p < 0.05; ^**^p ≤ 0.01 vs. the control or P1 condition.

The immunomodulatory effects of MSCs are jointly executed by both secretory factors and direct cell-to-cell contacts (Hoogduijn et al., 2010) and the *in vitro* inflammatory profile of human MSC has been previously analyzed (Schinköthe et al., 2008). Here we have determined the accumulation of several cytokines by MSC along the passages. Interestingly, MSC release low but constant levels of IL1β along the passages (≤200 pg/ml), exhibiting a significant induction after LPS plus IFN-γ challenge (at P2 and P3; Figure 5A). Regarding IL6 and IL8 levels, MSCs release higher levels of these cytokines which are strongly induced by the treatment with LPS or IFN-γ plus LPS up to P3, diminishing their levels to basal in successive passages (Figures 5B,C).

An additional aspect of the therapeutic potential of MSCs involves the capacity of these cells to extravasate after systemic administration that has been associated to the expression of MMP2/MMP9 (Ries et al., 2007) and which activity is controlled by the inhibitor proteins TIMP (De Becker et al., 2007). We determined that along the passages, MSCs express lesser levels of mRNA of MMP9 but significantly higher quantity of TIMP1 and TIMP2 (Figures 5D,E), suggesting that the migratory capacity rapidly drops after subculturing the cells, reducing the capacity to reach target tissues after inoculation. Moreover, the MMPs activity determined by zymography also exhibit an expansion-dependent decrease across passages (Figure 5F). According to this, the migratory capacity of MSCs determined in a “wound healing” assay was elevated in P1 but is significantly lower in P4 both at 24 h and 7 days (Figure 5G).

**Figure 6:**
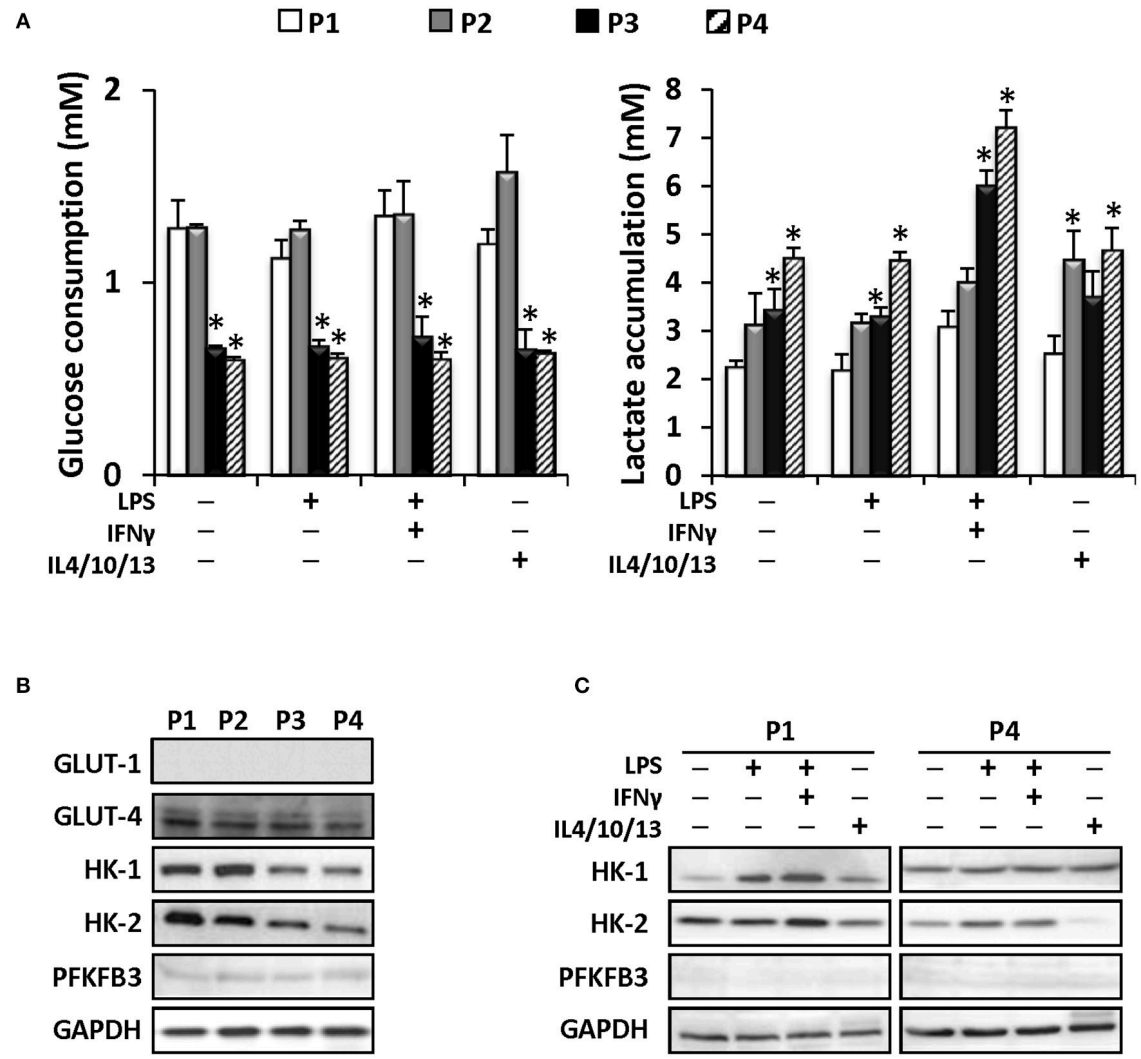
**MSCs efficiently metabolized glucose until the P4 producing higher levels of lactate in consequence**. MSC were treated as indicated and the glucose (left panel) and lactate (right panel) levels were determined in the supernatants from P1 to P4 **(A)**. Protein levels of several metabolic enzymes as GLUT-1, GLUT-4, HK-I, HK-II, and PFKFB3 were determined from P1 to P4 in control conditions **(B)**. MSCs were treated as indicated **(C)** and protein levels of HK-I, HK-II and PFKFB3 were analyzed in P1 and P4. GAPDH was used as a normalization protein in both panels. ^*^p < 0.05 vs. P1 condition.

Moreover, since metabolic rewiring is important in the functional responses of proliferating and immune cells (Pattappa et al., 2011), we have evaluated glucose consumption and lactate release by MSCs after confluence at each expansion cycle. As Figure 6A shows, MSCs metabolize glucose more actively at P1 and P2, whereas a drop at successive passages is observed. Regarding lactate, an increase in the ratio of lactate accumulation vs. glucose consumption is progressively observed from P1 to P4, indicating that MSCs are increasing anaerobic metabolism along the passages at the time that challenge with pro-inflammatory cytokines potentiates this metabolic shift regardless the passage (Figure 6A). Moreover, the levels of glucose transporters GLUT-4, HK-1, and HK-2 drop along the passages, in agreement with the decrease in glucose usage (Figure 6B), lacking the expression of the PFKFB3, a key enzyme in the positive regulation of the upper part of glycolysis (Mahlknecht et al., 2003). Indeed, the improvement of glucose consumption after pro-inflammatory challenge parallels the expression of HK-1 and -2 at P1 and P4, being PFKFB3 absent under these conditions (Figure 6C). These data indicate a significant shift in energy metabolism across the expansion of the MSC in culture.

## Discussion

Most of human tissues and organs do not regenerate spontaneously, justifying why cell therapy is today a significant issue in tissue and organ repair strategy, leading to the concept of “regenerative medicine” as an exciting new area of biomedical research (Bajada et al., 2008; Wu and Hochedlinger, 2011; Sykova and Forostyak, 2013). In this regard, adult MSCs represent an innovative tool for cell-based therapy of degenerative disorders (Joyce et al., 2010; Uccelli et al., 2011), chronic inflammation (González et al., 2009), autoimmune diseases (Constantin et al., 2009) and allograft rejection (Ringden et al., 2006). These cells have great plasticity but their physio-pathological properties remain poorly understood. Therefore, a better characterization of the mechanisms that mediate or modulate the therapeutic potency of MSCs is important from both physiological and clinical points of view.

MSCs have been considered an appealing source for cell therapy because they can be easily obtained and expanded *in vitro*. The number of clinical trials using MSCs has been rising since 2004; however, although the use of MSCs in clinical settings began with high enthusiasm in many countries, with China, Europe and US leading the way (http://clinicaltrial.cn), numerous scientific issues remain to be resolved before the establishment of clinical standards and governmental regulations (Wei et al., 2013). The heterogeneity of MSCs preparations *in vitro* has been considered as one of the major challenges that significantly impair progress in basic and translational MSC research as well as in the development of MSC therapies (Jones and Schäfer, 2015). Recent studies showed that the ability of expansion and differentiation of MSC may depend on several factors, such as donor's age, gender and *in vitro* cellular aging on the phenotypic, functional and molecular characteristics of MSCs (Brooke et al., 2008; Katsara et al., 2011). In this context it is essential to determine the maximal number of replications that are possible to maintain MSCs in culture without loss of stem and regenerative properties. Our results indicate that MSCs obtained from human bone marrow of healthy donors, maintain their stem properties only in the first passages, with specific and reliable differential responses from P1 to P4 passages, losing their pluripotency in the subsequent expansions. As an example, we have observed a better capacity of differentiation into a “myogenic-like” phenotype at P1. In addition, these cells express several “stemness” markers, such as c-Myc, KLF4 and Nanog, but the levels of some of these proteins diminished significantly with the successive passages suggesting that these cells lose their growth capacity after prolonged culture. Special mention deserve KLF4 and c-Myc that, perhaps after forcing the cells to proliferate, are progressively over-represented in the P1–P4 expansion. Thus, we propose that MSCs must not be replicated more than two or three passages *in vitro* before using in regenerative medicine since this fact ensures the maintenance of pluripotency and, perhaps favors a positive interaction with the damaged tissue that determined the commitment of the engrafted MSC.

Another critical aspect to be addressed in MSCs physiology is the immunological response of these cells by itself, which is basic to understand their response to several factors that can be present in the damaged tissue scenario. We have determined that depending on the stimulation, these cells presented higher protein levels of NOS-2, HO1 and Arg1 as well as an increased release of inflammatory cytokines to the culture medium. This fact highlights that, despite its low immunogenicity, MSCs have certain capacity to respond according to the local immune context. Indeed, the capacity to respond to classic pro-inflammatory (LPS and IFN-γ) and anti-inflammatory and pro-resolution factors (IL4, IL10, and IL13) of “professional” immune cells (Rodriguez-Prados et al., 2010) is retained only during P1 and P2 cycles. Although it is not possible to preclude the microambience to which these cells are confronted during therapeutic administration, the fact that classic immunomodulators, such as the prostanoids derived from cyclooxygenase 2 (i.e., PGE_2_) also accumulate across cell expansions, it is possible to suggest that immunotolerance progresses when the cells are used at passages higher than 2. Regarding metabolic adaptations, it has been shown that the initial induction of human and mouse pluripotent stem cell differentiation is associated to enhanced glycolysis but driven to production of acetyl-CoA and acetate, in addition to lactate (Moussaieff et al., 2015). Here we show that at late passages (P3–P4), glucose consumption is decreased, but lactate released is enhanced suggesting in impaired use of these substrates for TCA and acetate synthesis. In agreement with these results, the lesser glycolytic usage may trigger a loss of acetate synthesis and histone acetylation, reducing the capacity to maintain a pluripotent state (Hussein et al., 2014; Moussaieff et al., 2015). Moreover, we have proved that MSCs have a high migratory potential in the first passages, mainly associated to MMP2 activity, but this capacity rapidly declines after successive passages. This fact may be related to the significant decrease in the expression of MMPs as well as an augmentation of TIMP1 and TIMP2 levels from P2 in advance that are associated to a decreased collagenase activity of MMPs. These observations need to be considered when modulation of the migratory capacity of the MSCs is a central issue in the specific application for regenerative purposes since, after passage 2, the ability to migrate is negligible.

In addition to this, our data suggest that the glucose metabolism of MSC also experiences significant changes upon culture, moving toward a more anaerobic phenotype: whereas glucose consumption decreases after P2, the release of lactate increases suggesting a lesser capacity to use glucose through the TCA cycle. Although it can be proposed that histone acetylation is decreased under these conditions, the significance of this shift is at present unknown and further work is necessary to evaluate how energy metabolism changes after *in vitro* expansion of the cells, but the possibility exists to manipulate this central determinant of cell fate (i.e., under hypoxic conditions, restricting glucose, fatty acids and amino acid metabolism) to improve the regenerative capacity of MSC. Nevertheless, before MSC-based therapeutics can be applied in the clinic, more research is necessary to understand their behavior upon transplantation as well as the mechanisms of their interaction with the diseased microenvironment (Stoltz et al., 2015). The enormous advantages that offer the MSC for cell and/or tissue replacement after transplantation (autologous administration, *in vitro* manipulation, etc.), and their prolonged clinical use raises heavy debates in the fields of tissue engineering and regenerative medicine to date. Unraveling specific applications of this the therapeutic option may open new ways in this area, but a better understanding of their basic biology is essential to provide the rationale to these approaches.

## Materials and methods

### Materials

LPS was from Sigma-Aldrich, IFN-γ, IL4, IL10, IL13 were from PeproTech. 5-Aza-2′-deoxycytidine (5-Aza-C) was from Sigma-Aldrich. Antibodies were from Santa Cruz Biotech, Ambion, Millipore, Abcam and Cell Signaling Tech (see Table 1). PCR probes were from Invitrogen. Fluorescent secondary antibodies were from Molecular Probes. Tissue culture dishes were from Falcon and tissue culture media were from Gibco-Invitrogen.

**Table 1:** **Antibodies used in western blot and immunofluorescence assays**.

**Antibody**	**Supplier**	**Number**	**Working use**
Arg1	Santa cruz biotechnology	sc-20150	1:1000
B-MHC	Abcam	ab15	1:1000
c-Myc	Cell signaling technologies	5605	1:1000
COX-2	Santa cruz biotechnology	sc-1747	1:500
cTnT	Abcam	ab10214	1:1000
GAPDH	Ambion	AM4300	1:20,000
GLUT-1	Abcam	Ab40084	1:1000
HK-1	Cell signaling technologies	2014	1:1000
HO1	Millipore	AB1248	1:2000
KLF4	Cell signaling technologies	4038	1:1000
NANOG	Cell signaling technologies	4903	1:1000
NOS-2	Santa cruz biotechnology	sc-650	1:500
PFKFB3	Santa cruz biotechnology	sc-10091	1:500

### Human mesenchymal stem cell culture

Bone marrow samples were obtained from iliac crests aspirates over heparin from 11 healthy volunteers (6 men, 5 women) after informed consent, and were used in accordance with the procedures approved by the human experimentation and ethics committees of Hospital General Universitario Gregorio Marañón (Madrid). The mean of age was 50.5 ± 19.6 years. Mononuclear cells were isolated by centrifugation through 1.073 g/ml Ficoll at 1100 g for 30 min, rinsed twice with PBS, counted and seeded at 1.5 × 10^5^ cells per cm^2^ in complete medium (DMEM, 10% FBS, 5% HS, 100 U/ml penicillin and streptomycin) at 37°C in a humidified 5% CO_2_ air atmosphere. Three days later, non-adherent cells were removed by replacing the medium. After 10 days in culture, adherent cells formed homogenous colonies. The adherent cells were resuspended after trypsin treatment and re-plated at a density of 8,000/cm^2^ (approximately 1:3). The medium was changed every 3 days and these cells were considered as “multipotent mesenchymal stromal cells” (MSCs). MSCs expressed ≥95% of CD105, CD73, and CD90 and lacked the expression (≤2% positives) of CD45, CD34, CD14, CD11b, CD79α, CD19, and HLA class two, as analyzed by flow cytometry. Moreover, these cells differentiate into osteoblasts, adipocytes and chondroblasts under standard *in vitro* differentiation conditions following the criteria of the International Society for Cellular Therapy (ISCT) (Dominici et al., 2006). MSCs were grown and expanded for at least 4 passages in culture. Morphologically, MSCs were defined by a fibroblast-like appearance.

### Myogenic differentiation of MSCs *in vitro*

Cells were seeded in DMEM plus 10% FBS and exposed to 10 μM of 5-Aza-C for 24 h. To prevent cell death from prolonged 5-Aza-C exposure, cells were washed twice with PBS and the medium was replaced. Cells were maintained in the absence or 5-Aza-C for 5 days to allow the complete differentiation to cardiomyocytes.

### Preparation of total cell extracts

Cells were homogenized at 4°C in a medium containing 10 mM Tris-HCl, pH 7.5; 1 mM MgCl_2_, 1 mM EGTA, 10% glycerol, 0.5% CHAPS and proteases and phosphatases inhibitors cocktails (Sigma #P8340, #P5726, #P0044). The extracts were vortexed for 30 min at 4°C and after centrifuging for 15 min at 13,000 g, the supernatants were stored at −20°C. Protein levels were determined with Bradford reagent (Bio-Rad #500-0006).

### Western blot analysis

Equal amounts of protein (20–40 μg) were loaded and size-fractionated onto 8–12% SDS-PAGE and transferred to a PVDF membrane (Bio-Rad #170-4157). After blocking with 5% non-fat dry milk and incubation with the corresponding Abs (Table 1) the blots were developed by ECL protocol and different exposition times were performed for each blot to ensure the linearity of the band intensities. Values of densitometry were determined using Image J software.

### Immunofluorescence microscopy

Cells were seeded for 16–24 h into sterile 8-wells Chamber Slides (Falcon). After treatments, cells were fixed with 2% paraformaldehyde for 10 min, permeabilized in iced methanol and incubated with 3% BSA for 30 min (Sigma #A2153). After incubating with Ab against cardiac myosin, troponin T, c-Myc, KLF-4 and Nanog (see Table 1) at 4°C for 18 h, cells were washed with PBS followed by incubation with Alexa 488 anti-rabbit secondary antibody (1:500, Molecular Probes) and DAPI 1:1000 for 20 min. Coverslips were mounted in ProLong® Gold Antifade reagent (Life technologies #P36930) and examined using a confocal microscope Leica PCS SP5.

### Prostaglandin E_2_, glucose and lactate determinations

Prostaglandin E_2_ was measured in the culture media with a commercial kit from Arbor Assays, following the manufacturer's instructions. Glucose and lactate were determined using commercial kits from Biosystems and SpinReact, SA, following the manufacturer's instructions.

### Detemination of NO levels

The amount of nitrites in the culture medium was measured after reduction of nitrate to nitrite and quantification of nitrite using a previous protocol (Rodriguez-Prados et al., 2010)

### DIaplex human Th1/Th2/inflammation kit

The quantification of multiple human cytokines (IFN-γ, TNF-α, IL1β, IL2, IL4, IL6, IL8, IL10, IL12p70, and IL17A) was performed in culture supernatants by flow cytometry using a commercial kit from Diaclone (Gen-Probe) following the manufacturer's instructions. This is a multiplexed fluorescent bead-based immunoassay for the simultaneous quantification of multiple analytes from a single sample, utilizing bead populations with distinct fluorescence intensities. Once the data were acquired, we performed the data analysis using the specific DIAplex Pro software supplied by www.genprobe.com, as indicated in the website.

### Human interleukin ELISA kits

Data from diaplex were verified using specific human ELISA kits to quantify IL6 and IL8 (Abcam #ab46042 and #ab46032, respectively). These assays were carried out following the manufacturer's instructions.

### PCR

RNA was extracted from cells using TRI Reagent® solution (Ambion #AM9738) and 1 μg was reverse-transcribed into cDNA using the *Transcriptor first strand cDNA synthesis kit* (Roche #04897030001). Then, PCR reaction was performed with this template cDNA adding *FastStart Universal SYBR Green Master* (Roche #04913850001) and specific primers (Table 2) in a MyIQ thermocycler (Bio-Rad).

**Table 2:** **Primers used in qPCR analysis**.

**PRIMER**	**Forward**	**Reverse**
β-MHC	GGGATAACCAGGGGAAGCACCAAGA	ACTTGCGGAGGTACTGGGCCG
cTnT	AAGCCCAGGTCGTTCATGCCC	CCATGCGCTTCCGGTGGATGT
IL13Rα1	CCGCGCCTACGGAAACTC	CGGGTGGATTCCATGTCCATA
IL10R	CTGAAGAGCCCCAGTTCCTC	TCCCGCTGTCTGTGCTATTG
MMP2	TACAGGATCATTGGCTACACACC	GGTCACATCGCTCCAGACT
TIMP2	ACAGGCGTTTTGCAATGCA	GGGTTGCCATAAATGTCGTTTC

### Zymography assay

Supernatants were incubated for 1 h RT in loading buffer without reducing agents (50 mM Tris HCl pH6.8, 8% glycerol, 2% SDS, 0.004% bromophenol blue). After electrophoresis at 50 V ON 4°C in a resolving acrylamide gel (11% for MMP2 and 7.5% for MMP9) containing 1 mg/ml gelatin, gel was incubated in activation buffer (50 mM Tris-HCl pH 7.4, 0.2 M NaCl, 5 mM CaCl_2_, 1% TritonX100, 0.02% NaN_3_) at 37°C ON to allow that renatured MMPs in the gel digest their substrates. After incubation, the gel was stained with 0.5% Comassie® blue for 30 min RT and then washed with 30% ethanol/10% acetic acid solution for at least 1 h RT. Finally MMPs were detected as clear bands against a blue background of undegraded substrate that can be quantified by densitometry using Image J software.

### “wound healing” migration assay

Cells were seeded and culture until confluence. Using a pipette tip make a straight scratch, simulating a wound. Then, several images of the same area were taken at the beginning (time 0) and after 24 h and 7 days during migration to detect cells closing the wound. The migration rate was quantified using Image J software.

### Statistical analysis

The values in graphs correspond to the mean ± SD. The statistical significance of differences between mean sample values was estimated with a Student's *t*-test for unpaired observations. A p≤0.05 value was considered as statistically significant.

## Author contributions

PP, PS, FF, and LB conceived and designed the experiments, PP, MFV, MFS, and VT performed the experiments, PP and LB analyzed the data and PP, MFV, PM, and LB wrote the paper.

## Funding

This work was funded by grants SAF2014-52492R and RTC2015-3741 from MINECO; RD12/0042/0019 and Ciberehd are funded by the Instituto de Salud Carlos III, and FEDER.

### Conflict of interest statement

The authors declare that the research was conducted in the absence of any commercial or financial relationships that could be construed as a potential conflict of interest.
